# Potential benefit of bosentan therapy in borderline or less severe pulmonary hypertension secondary to idiopathic pulmonary fibrosis—an interim analysis of results from a prospective, single-center, randomized, parallel-group study

**DOI:** 10.1186/s12890-017-0523-2

**Published:** 2017-12-13

**Authors:** Yosuke Tanaka, Mitsunori Hino, Akihiko Gemma

**Affiliations:** 1 Department of Respiratory Medicine, Nippon Medical School, Chiba Hokusoh Hospital, 1715 Kamagari, Inzai, Chiba 270-1694 Japan; 2 Department of Pulmonary Medicine and Oncology, Graduate School of Medicine, Nippon Medical School, 1-1-5 Sendagi, Bunkyo-ku, Tokyo, 113-8603 Japan

**Keywords:** Pulmonary hypertension, Idiopathic pulmonary fibrosis, Right heart catheterization, Echocardiography, Endothelin receptor antagonists

## Abstract

**Background:**

No drugs have been approved for the treatment of patients with pulmonary hypertension (PH) secondary to idiopathic pulmonary fibrosis (IPF), particularly those with idiopathic honeycomb lung. This study was conducted to investigate the long-term efficacy and safety of bosentan for PH based on changes in prognosis and respiratory failure.

**Methods:**

IPF patients with borderline or less severe PH and completely organized honeycomb lung were randomized (1:1) to bosentan or no treatment for PH for 2 years and assessed at baseline and every 6 months for respiratory failure, activities of daily living (ADL), lung and heart functions by right cardiac catheterization, and other parameters. An interim analysis was performed, however, following detection of a significant survival benefit favoring bosentan therapy.

**Results:**

Significant differences were noted for the bosentan-treated (*n* = 12) vs. untreated (*n* = 12) groups in hospital-free survival (603.44 ± 50.074 days vs. 358.87 ± 68.65 days; hazard ratio [HR], 0.19; *P* = 0.017) and overall survival (671 days vs. 433.78 ± 66.98 days; HR, 0.10; *P* = 0.0082). Again, significant improvements were noted for the bosentan-treated group from baseline to month 6 or 12 in several indices in ADL, pulmonary circulation, and %DLCO. Without requiring O_2_ inhalation, bosentan was associated with no increase but a trend toward a decrease in adverse events and an improvement in respiratory status.

**Conclusions:**

Bosentan tended to improve prognosis and ADL without worsening respiratory failure in IPF patients with borderline or less severe PH and completely organized honeycomb lung alone.

**Trial registration:**

This study was registered on December 18, 2010 with UMIN-CTR Clinical Trial as UMIN000004749 to investigate the long-term influence of bosentan on cardiac function, as well as its cardioprotective efficacy and safety, in patients with pulmonary hypertension secondary to concurrent COPD and IPF, respectively.

**Electronic supplementary material:**

The online version of this article (doi: 10.1186/s12890-017-0523-2) contains supplementary material, which is available to authorized users.

## Background

Idiopathic pulmonary fibrosis (IPF) is a disorder associated with poor prognosis. Pulmonary arterial hypertension (PAH), which is likely to lead to right heart overload, is also associated with poor prognosis. Patients with IPF are at risk of developing pulmonary hypertension (PH) as the underlying condition worsens or becomes severer, thus further compromising their prognosis [1–8]. However, it remains unclear how long it may typically take for patients with IPF to start developing PH or for its associated influence on cardiac function to become manifest [1]. Given that, once elevated, pulmonary arterial pressure (PAP) becomes irreversible because of established vascular remodeling [1, 9], it appears that efficacious treatment should be started before its onset. Furthermore, it is assumed that cardiac overload starts even before the onset of PH. However, to date, no drugs have been approved for the treatment of PH secondary to respiratory diseases, such as IPF [1].

Drugs specific for PAH, such as bosentan (Tracleer Tablets®), have been reported in some studies to effectively improve PH in patients with respiratory diseases, such as COPD and IPF [1, 10–13].

While the results of randomized controlled studies conducted to date, such as BUILD-1 and BUILD-3 studies [11], appear to argue against the use of bosentan in patients with pulmonary hypertension (PH), these studies involved a wide range of patients from those with fibrotic idiopathic interstitial pneumonia (f-IIP) and fibrotic nonspecific interstitial pneumonia (f-NSIP) to those with highly elevated pulmonary arterial pressure (PAP) and decreased cardiac index (CI), where the presence of multiple risk factors for IPF in these patients may have additively or synergistically contributed to the therapeutic outcomes reported in these studies. Again, the benefit of early intervention with bosentan may not have been sufficiently explored in those with mildly elevated PAP in these trials, while bosentan was indeed shown to be efficacious against IP in a subset of patients in the BUILD-1 study, despite the observation that many patients with IPF are associated with rapidly elevated PAP as well as progression of IPF and that elevated PAP is associated with poor prognosis [7].

Against this background, the present bosentan study focused on IPF patients with completely organized honeycomb lung without any pulmonary (including IP) lesions who chiefly complained of symptoms suggestive of progressive respiratory failure, i.e., progressive dyspnea with minimal IPF activity.

Thus, the IPF patients with completely organized honeycomb lung alone were enrolled in this study to ensure that the subjects in this study had pathologically stable IPF and required regular hospital visits for treatment. An interim analysis was performed, however, in patients with borderline or less severe PH (25 mmHg ≤ mean pulmonary arterial pressure [mPAP] at rest <35 mmHg and/or mPAP on effort [mPAPOE] ≥ 30 mmHg), following detection of a greater-than-expected significant survival benefit in patients with borderline or less severe PH treated with bosentan at an early phase of the trial when the number of patients enrolled was still small.

## Methods

### Study design and methods

This was a prospective, single-center, interventional, parallel, randomized, open-label study.

### Target patient population

This study was conducted in patients with IPF (WHO functional class II, III or IV) who showed no signs of hypoxia during 6-min walk test (6MWT) and therefore had enough functional capacity for ADL and who gave written informed consent to participate in the study.

### Eligibility criteria

To be included in this study, patients had to fulfill all of the following inclusion criteria but none of the following exclusion criteria:

### Inclusion criteria

1. Patients aged 20 years old or older (both sexes)
2. Patients diagnosed at this hospital as having IPF (WHO functional class II, III or IV) without hypoxia at rest or during 6MWT (to exclude those with decreased ADL and dyspnea in daily living associated with hypoxia and to minimize the influence of hypoxic pulmonary vasoconstriction [HPV] as a potential cause of PH associated with decreased partial pressure of oxygen in arterial blood [PaO_2_]) (PaO_2_ < 60 mmHg)*.
  *Including those whose hypoxia (PaO_2_ < 60 mmHg) had been corrected with long-term oxygen therapy (LTOT)
3. Patients with stable IPF who had not required any change of treatment within 3 months prior to study entry, i.e., those confirmed to have completely organized honeycomb lung and no active inflammatory lesion, such as grand glass opacity (GGO) (chronic IIP based on high-resolution computed tomography (CT) findings for which no effective therapy exists); and who presented to our hospital for the first time with symptoms of progressive respiratory failure and had not received any medical treatment for IPF within 3 months prior to their visit.
  *Excluding those whose progressive respiratory failure required no treatment for IPF itself and those who had an increased LTOT dose as a minimum requirement for progressive respiratory failure.
4. Inpatients and outpatients
5. Patients who provided written informed consent to participate in this study

### Exclusion criteria

1. Patients who had received bosentan or any other drug specific for PAH (e.g., phosphodiestetrase type 5 [PDE-5] inhibitors, endothelin receptor antagonists, or prostaglandin analogs) prior to their enrollment
2. Patients with any disease that could cause right heart overload
3. Patients with hypoxia during 6MWT (PaO_2_ < 60 mmHg)*.
  - Excluded were those whose hypoxia (PaO_2_ < 60 mmHg) had been corrected with LTOT (i.e., those in whom LTOT is in place to ensure PaO_2_ > 60 mmHg both at rest and during 6MWT, who were deemed equivalent to IPF patients receiving routine therapy in clinical practice to allow them to be monitored for changes in their condition, prognosis and functional capacity for ADL).
4. Women who were pregnant or might have been pregnant, and who were lactating
5. Patients with moderate or severe liver disorder
6. Patients receiving treatment with cyclosporine, tacrolimus, or glibenclamide
7. Other patients judged by the investigator to be ineligible for this study (e.g., those with any disease or condition other than IPF that might affect their ADL, such as arrhythmia, LV failure, pulmonary thromboembolism, connective tissue diseases, intervertebral disc herniation, as they were confirmed by history taking, physical examination, chest x-ray, echocardiography [ECG], lung perfusion scintigraphy, and measurements of various parameters conducted during the run-in period).

### Grouping of patients

In order to include those with minimal IPF activity alone, of all patients first diagnosed with IPF at our hospital based on the presence of chronic f-IIP as confirmed by high-resolution CT findings, those whose chief complaints suggested progressive respiratory failure and who were suffering from progressive dyspnea were evaluated for PAP by right heart catheterization (RHC) and ECG, as well as for right heart function by ECG.

According to the current diagnostic criteria, if mPAP at rest is <25 mmHg, the patient is not diagnosed as having PH even if the mPAPOE is ≥30 mmHg. In our study, however, this state was defined as borderline PH representing a very mild form of PH; and besides, 25 mmHg ≤ mPAP at rest <35 mmHg was defined as less severe PH. Since the aim of this study was to evaluate the efficacy and safety of early therapeutic intervention with bosentan in PH, borderline PH or PH was diagnosed if mPAP at rest was ≥25 mmHg and/or mPAPOE was ≥30 mmHg and mPAWP was ≤15 mmHg. Moreover, borderline PH or less severe PH was defined as mPAP <25 mmHg and mPAPOE ≥30 mmHg or 25 mmHg ≤ mPAP <35 mmHg; severe PH was defined as mPAP ≥35 mmHg; and non-borderline PH or PH was defined as non-PH (mPAP <25 mmHg and mPAPOE <30 mmHg).

#### Drug-treated and untreated patients

All patients who met the eligibility criteria and gave informed consent to participate in this study were evaluated for PAP and right heart function and those with mPAP at rest ≥25 mmHg and/or mPAPOE ≥30 mmHg were stratified by mPAP: mPAP at rest <35 mmHg vs. ≥ 35 mmHg. Patients in each subgroup were randomly allocated to either bosentan (drug-treated group) or no treatment (untreated group) by the envelope method. Patients diagnosed with non-PH (mPAP at rest <25 mmHg with mPAPOE <30 mmHg) were assigned to the untreated group.

The drug-treated group comprised those who were diagnosed at this hospital as having IPF without hypoxia (PaO_2_ > 60 mmHg) and who gave informed consent to participate in this study after PAP and right heart function assessments.

**Fig. 1:**
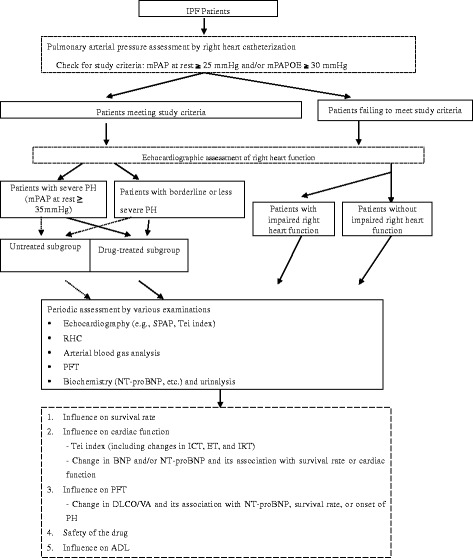
Patient flowchart

The untreated group comprised those diagnosed at this hospital as having IPF without hypoxia (PaO_2_ > 60 mmHg) and who gave their informed consent to participate in this study after PAP and right heart function assessments. This group included those with severe PH (mPAP at rest ≥35 mmHg), borderline or less severe PH (25 mmHg ≤ mPAP at rest <35 mmHg and/or mPAPOE ≥30 mmHg), and non-PH (without borderline PH or PH). (Fig. 1; see also Additional file 1).

An interim analysis was performed, however, in patients with borderline or less severe PH (25 mmHg ≤ mPAP at rest <35 mmHg and/or mPAPOE ≥30 mmHg), following detection of a greater-than-expected significant survival benefit in patients with borderline or less severe PH treated with bosentan, at an early phase of the trial when the number of patients enrolled was still small.

The study required that IPF patients be randomized to drug-treated and untreated groups to investigate their clinical course in real-world settings, with no change of treatment allowed including bosentan for 2 years or until their death as a rule, except for minimal symptomatic therapy (including oxygen volume adjustments required to ensure similar oxygen conditions among the patients), which met none of the exclusion criteria.

### Target sample size

See Additional file 2.

### Outcome measures

ECG examinations were carried out during the run-in period* and every 6 months thereafter**. Complete two-dimensional, pulsed-wave, color-flow echocardiography was performed using the Toshiba ultrasound system Xario (TOSHIBA MEDICAL SYSTEMS CORPORATION, Tochigi, Japan) as previously described [14–25]. (See also Additional file 3).

Doppler measurements were carried out during the run-in period* and every 6 months**. (See also Additional file 3 and Additional file 4: Fig. S1).

RHC was carried out during the run-in period* and every 6 months thereafter**. Hemodynamic parameters (systolic PAP [SPAP]; diastolic PAP [DPAP]; mean PAP [mPAP]; systolic PAWP [SPAWP]; diastolic pulmonary capillary wedge pressure [DPAWP]; mean PAWP [mPAWP]; systolic right ventricular pressure [SRVP]; diastolic RVP [DRVP]; mean RVP [mRVP]; systolic right atrial pressure [SRAP]; diastolic RAP [DRAP]; mean RAP [mRAP]; and cardiac output [CO]) and pulmonary vascular resistance (PVR) were measured, with the patient in the supine position, via the internal jugular vein and using a Swan-Ganz continuous cardiac output (CCO) thermodilution flow-directed pulmonary artery catheter (Edwards Lifesciences LLC, USA). Cardiac output was measured by the thermodilution method using a Vigilance hemodynamic monitor (Edwards Lifesciences LLC, USA).

Systolic PAP on effort (SPAPOE), diastolic PAP on effort (DPAPOE) and mean PAP on effort (mPAPOE) were measured while patients were clasping and opening both hands repeatedly by putting a full strain on the body. Furthermore, mixed venous blood gas analysis was performed.

### Survival analysis

Hospital-free survival and overall survival were determined by the duration of survival from week 0 (start of assessment), i.e., as the date treatment started for the drug-treated group and 2 weeks after RHC for the untreated group. Even for those unable to undergo the periodic assessments due to change of their attending physician, etc., this survival analysis was continued by contacting the patient’s current physician to have his/her survival status confirmed. Patients were censored from hospital-free survival if they could no longer continue ambulatory treatment and were admitted to another hospital or if they could no longer present to our hospital for progression of respiratory failure.

### Adverse events

All patients were assessed for adverse events during the run-in period* and every 4 weeks thereafter**, as well as based on their medical records on unscheduled visits to our outpatient clinic. Even for those unable to undergo the periodic assessments due to change of their attending physician, adverse events were assessed by contacting their current physician to have these events confirmed.

### Other parameters

Pulmonary function test (PFT) was carried out during the run-in period* and every 6 months thereafter**. (See also Additional file 3).

ADL assessments including exercise tolerance test [26–28] were performed during the run-in period* and every 6 months thereafter**. (See also Additional file 3 and Additional file 5: Figure S2). Those in whom LTOT was in place to ensure adequate oxygen inhalation during 6MWT (deemed equivalent to IPF patients receiving routine therapy in clinical practice to allow them to be monitored for changes in their condition, prognosis and functional capacity for ADL) were assessed for treadmill exercise test (TMET) with LTOT in place.

Arterial blood gas (ABG), arterial plasma lactate, brain natriuretic peptide (BNP) and N-terminal (NT)-proBNP were determined during the run-in period and every 6 months thereafter. [29] (See also Additional file 3).

Hematology, biochemistry and urinalysis were performed during the run-in period* and every 4 weeks thereafter**.

*Run-in period: Within 2 weeks after written informed consent was obtained from each patient.

**Every 6 months: Consecutive 6 months with a ± 1-week window counting from week 0 (start of assessment) defined as the date drug treatment started for the drug-treated group and 2 weeks after RHC for the untreated group. However, the periodic assessments not conducted in patients as planned based on the attending physician’s judgement were deemed acceptable, unless they met any of the criteria for discontinuation of the study (e.g., pneumonia, etc.) (Fig. 1 and Additional file 6: Figure S3).

### Study drug

Bosentan was administered, as a rule, according to the approved dosage and administration. Bosentan is to be usually initiated in adults at a dose of 62.5 mg twice daily after breakfast and dinner for 4 weeks and increased to a dose of 125 mg twice daily after breakfast and dinner from week 5 of treatment onwards with the dosage adjusted according to the patient’s symptoms and tolerability, but not exceeding 250 mg per day. In this study conducted in routine clinical settings, however, it was acceptable to continue treatment at the initial dosage if deemed by the investigator to be appropriate based on the patient’s condition (see also Additional file 7).

### Concomitant drugs and therapies

Drugs allowed for use in the study included drugs intended for the treatment of the underlying disease (IPF) and drugs, other than drugs specific for PAH, for the treatment of PH as required for aggravation of PH. Drugs prohibited for use included cyclosporine, tacrolimus, glibenclamide and other drugs specific for PAH (e.g., PDE-5 inhibitors, endothelin receptor antagonists and prostaglandins) as well as any other investigational drug.

### Study period

The study was conducted for 24 months between September 2010 and September 2022 with patient enrollment lasting until January 2020 (see Additional file 8).

### Statistical analysis

Data are expressed as the mean ± standard deviation (SD). Changes from baseline in individual outcome measures were compared between drug-treated and untreated patients, and analyzed for statistical significance. Analysis on paired data was performed using Mann-Whitney U test. Changes in trend over time were analyzed using the Residual Maximum Likelihood (REML) or least squares method. All statistical analyses were performed using JMP version 11.2.1 (SAS Institute Inc., Cary, NC). A two-sided *P* value of <0.5 was considered to indicate a statistically significant change.

## Results

### Patients

This report presents the results of an interim analysis of the IPF patients in this study. A total of 32 IPF patients were enrolled in this study between February 2011 and June 2016, who comprised all the outpatients who had met the study entry criteria. At the time of their initial presentation to our hospital, all patients were confirmed to have chronic fibrotic idiopathic interstitial pneumonia (IIP) based on high-resolution CT findings of completely organized honeycomb lung with basal predominance in bilateral subpleural regions for which no effective therapy exists. Patients chiefly complained of symptoms of progressive respiratory failure. While all patients confirmed to have no progressive pulmonary fibrosis on CT were given detailed explanations as to the potential adverse effects associated with the use of antifibrotics, such as pirfenidone or nitentanib, which has only recently been launched in Japan and indicated for very few patients, as well as the costs due under current health insurance, none were confirmed to have received any treatment specific for IPF (e.g., pirfenidone or nintentanib) within 3 months prior to their enrollment or wished to receive any antifibrotic drug after the first 3 months or later, and none dropped out because of any treatment given, other than symptomatic treatment, for PH as the underlying disease, such as calcium channel blockers.

Of these 32 patients, the following 3 patients were excluded from the study: 1 who was found to have cancer during the study, which had probably existed at the time of enrollment (untreated, borderline or less severe PH group), 1 who developed symptoms of disc hernia during the run-in period (non-PH group), and 1 who died from aspiration pneumonia during the run-in period before the start of bosentan therapy (drug-treated, borderline or less severe PH group). The remaining 29 patients who had completed the study or were still on the study treatment were included in the present analyses. Of these 29 patients, 3 (including 1 female) had no borderline PH or PH (non-borderline PH/PH) and the remaining 26 patients with boarderline or less severe PH, or severe PH were randomized to receive or not to receive bosentan therapy. Of these, 13 were in the drug-treated group and the other 13 were in the untreated group, including 1 in each group confirmed to have mPAP at rest ≥35 mmHg (severe PH), and 12 in the drug-treated group (age range, 56–76 years old) and 12 in the untreated group (age range, 51–80 years old) confirmed to have mPAP at rest <35 mmHg (borderline or less severe PH).

Patient demographics and characteristics were similar between the untreated, borderline or less severe PH group and the drug-treated, borderline or less severe PH group (Table 1).

**Table 1:** Clinical characteristics of subjects with borderline or less severe PH (mPAP <35 mmHg)

	Untreated borderline or less severe PH	Drug-treated borderline or less severe PH	*P* ^*^
No. (male/female)	12(8/4)	12(9/3)	0.66
Age (y.o.)	70.50 ± 7.97	66.92 ± 6.45	0.11
Height (cm)	160.04 ± 10.11	160.87 ± 10.07	0.84
Weight (kg)	62.067 ± 12.17	54.95 ± 12.72	0.25
No. of patients with LTOT	8	7	0.67
ADL including exercise tolerance test			
WHO functional class	2.67 ± 0.78	2.83 ± 0.83	0.78
mMRC score	2.42 ± 1.084	2.33 ± 1.44	0.98
SGRQ score			
Symptoms	56.10 ± 22.87	45.78 ± 28.96	0.52
Activity	61.60 ± 22.35	55.18 ± 33.21	0.98
Impact	37.53 ± 23.11	34.38 ± 22.18	0.91
Total	49.42 ± 21.27	43.93 ± 26.41	0.77
SF36			
Physical functioning (PF)	45.83 ± 21.41	60.42 ± 26.41	0.11
Role physical (RP)	38.58 ± 21.96	51.058 ± 39.16	0.56
Bodily pain (BP)	72.17 ± 26.30	80.00 ± 25.23	0.45
General health (GH)	40.67 ± 18.34	46.75 ± 20.067	0.49
Vitality (VT)	49.34 ± 20.55	58.36 ± 29.23	0.40
Social functioning (SF)	56.25 ± 26.38	68.75 ± 33.50	0.35
Role emotional (RE)	62.51 ± 30.048	67.36 ± 37.69	0.52
Mental health (MH)	63.75 ± 19.67	65. 00 ± 27.88	0.62
Right heart cardiography			
mPAP (mmHg)	20.83 ± 5.75	21.17 ± 7.73	0.93
mPAPOE (mmHg)	42.67 ± 12.78	42.58 ± 8.87	0.45
mPAWP (mmHg)	6.83 ± 3.79	6.28 ± 3.57	0.76
mRVP (mmHg)	14.42 ± 3.58	14.083 ± 6.57	0.31
mRAP (mmHg)	2.50 ± 1.68	3.00 ± 2.13	0.45
CO (L/min)	4.80 ± 1.12	5.10 ± 1.27	0.82
CI (L/min/m^2^)	2.90 ± 0.56	3.21 ± 0.63	0.38
PVR (wood)	3.12 ± 1.65	3.022 ± 2.0031	0.95
PVRI	5.073 ± 2.67	4.58 ± 2.61	1.00
Mixed venous			
PHv	7.39 ± 0.029	7.40 ± 0.026	0.45
PvCO_2_ (mmHg)	49.34 ± 6.036	48.15 ± 4.22	0.82
PvO_2_ (mmHg)	36.69 ± 3.89	37.55 ± 3.93	0.60
SVO_2_ (%)	68.77 ± 5.47	70.53 ± 5.71	0.66
PFT			
%VC (%)	68.34 ± 16.92	69.55 ± 22.62	0.98
FVC (L)	2.0033 ± 0.57	2.087 ± 0.80	0.86
%DLCO (%)	30.72 ± 16.0019	27.37 ± 23.76	0.25
TTE			
ET (msec)	299.71 ± 55.95	263.83 ± 36.16	0.18
PAAcT (msec)	98.67 ± 32.65	94.75 ± 11.65	0.58
AcT/ET	0.33 ± 0.087	0.37 ± 0.060	0.23
PEP (msec)	92.71 ± 12.65	87.42 ± 18.62	0.12
ICT (msec)	17.083 ± 19.96	21.75 ± 21.73	0.70
IRT (msec)	55.71 ± 45.0023	52.42 ± 36.49	0.95
ICT + IRT (msec)	87.79 ± 64.54	72.82 ± 46.34	0.69
TEI index	0.32 ± 0.27	0.30 ± 0.25	0.98
TAPSE(cm)	2.32 ± 0.47	2.27 ± 0.55	0.75
Diastolic RA area (cm^2^)	8.20 ± 3.21	10.64 ± 4.91	0.29
Diastolic RA major axis (cm)	4.35 ± 2.0037	4.20 ± 2.018	0.66
Systolic RA area (cm^2^)	4.74 ± 2.10	5.46 ± 2.60	0.60
Systolic RA major axis (cm)	2.95 ± 0.99	2.65 ± 0.55	0.25
Diastolic RV area (cm^2^)	16.090 ± 6.57	15.39 ± 7.96	0.33
Diastolic RV major axis (cm)	6.38 ± 1.28	6.22 ± 1.098	0.49
Systolic RV area (cm^2^)	9.27 ± 3.47	9.38 ± 4.38	0.64
Systolic RV major axis (cm)	4.98 ± 1.42	4.95 ± 0.89	0.60
RVEF (%)	58.91 ± 12.45	51.98 ± 13.29	0.13
Aortic Blood data at rest			
pH	7.41 ± 0.027	7.42 ± 0.022	0.25
PO_2_ (mmHg)	76.84 ± 10.091	82.46 ± 7.93	0.11
Aortic oxygen saturation (%)	95.02 ± 1.55	95.85 ± 1.20	0.12
BNP (pg/ml)	29.42 ± 20.26	20.76 ± 13.10	0.34
NT-proBNP (pg/ml)	93.33 ± 60.15	69.67 ± 48.21	0.45
LA (mg/dl)	11.82 ± 4.082	10.00 ± 3.53	0.33
TMET			
METS	3.55 ± 1.89	3.96 ± 2.54	0.81
Post-TMET Aortic Blood data			
Post-TMET pH	7.34 ± 0.061	7.36 ± 0.069	0.45
Post-TMET PCO_2_ (mmHg)	46.94 ± 12.22	43.017 ± 6.75	0.66
Post-TMET PO_2_ (mmHg)	54.075 ± 15.93	67.23 ± 14.71	0.18
Post-TMET oxygen-Sat (%)	80.35 ± 18.077	90.85 ± 4.42	0.14
Post-TMET BNP (pg/ml)	40.20 ± 34.88	35.62 ± 46.66	0.27
Post-TMETNT-proBNP (pg/ml)	102.83 ± 67.48	108.67 ± 124.95	0.64
LA post TMET – LA at rest (mg/dl)	24.68 ± 20.012	22.82 ± 18.88	0.98
6MWD	246.18 ± 104.27	296.63 ± 128.0090	0.31
Post-6 MW Aortic Blood data			
Post-6MWT pH	7.39 ± 0.021	7.40 ± 0.039	0.15
Post-6 MW-PCO_2_ (mmHg)	41.042 ± 8.32	42.64 ± 5.42	0.53
Post-6 MW-PO_2_ (mmHg)	77.20 ± 30.98	72.067 ± 15.79	0.91
Post-TMET Oxygen-Sat (%)	92.58 ± 4.13	90.00 ± 8.35	0.69
Post-6 MW-BNP (pg/ml)	34.52 ± 25.66	25.080 ± 23.95	0.33
Post-6 MW-NT-proBNP (pg/ml)	98.50 ± 75.13	80.67 ± 72.41	0.47
LA post-6 MW – LA at rest (mg/dl)	8.60 ± 8.31	5.42 ± 8.34	0.14

Data presented as mean ± SD

^*^ *P* value for Mann-Whitney U test to assess the difference between the untreated and drug-treated patients with borderline or less severe PH

### Adverse events (Table 2)

#### Exacerbation of subjective symptoms of dyspnea (Table 2, Figure 2a)

Of the 12 untreated patients with borderline or less severe PH, 7 were confirmed to have experienced exacerbation of subjective symptoms of dyspnea based on the data obtained at the cut-off date, with the time to exacerbation of dyspnea being 152.00 ± 89.94 days (mean ± SD**)**. Of 12 the drug-treated patients with borderline or less severe PH, 3 were confirmed to have experienced exacerbation of dyspnea based on the data obtained at the cut-off date, with the time to exacerbation being 259.00 ± 49.87 days (mean ± SD**)**. Proportional hazard analysis showed that the risk ratio of the drug-treated group to the untreated group was 0.32, but with no significant difference.

**Table 2:** Adverse events observed in untreated and drug-treated patients with borderline or less severe PH

	Untreated borderline or less severe PH	Drug-treated borderline or less severe PH
Exacerbation of dyspnea	7	3
Time to exacerbation of dyspnea **(**mean ± SD**)** (days)	152.00 ± 89.94	259.00 ± 49.37
Increase of the O_2_ dose	5	2
Time to O_2_ dose increase **(**mean ± SE**)** (days)	199.00 ± 132.90,	335.00 ± 182.43
Decrease of the O_2_ dose	0	1
Hospitalization (hospital-free survival)	8 (241.50 ± 192.24)	2 (239.002 ± 169.00)
Death (survival)	7 (309.29 ± 195.13)	1 (671)
Other adverse events	3^a^	6^b^

^a^ Gastrointestinal hemorrhage (*n* = 1), pneumonia (*n* = 1), and ileus (*n* = 1)

^b^ Pneumothorax (*n* = 3), CHF (*n* = 2), and liver dysfunction (*n* = 1)

**Fig. 2:**
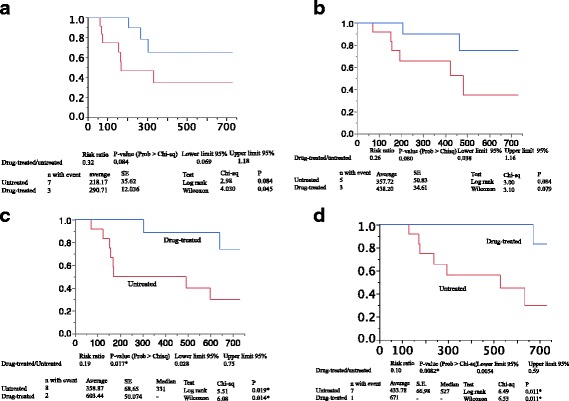
Analysis of survival by adverse event. **a** Analysis of the time to exacerbation of subjective dyspnea. Among the untreated patients with borderline or less severe PH, the time to exacerbation of dyspnea was 152.00 ± 89.94 days (mean ± SD) in 7 of 12 patients confirmed to have experienced exacerbation of subjective symptoms of dyspnea by the data obtained on the cut-off date. Among the drug-treated patients with borderline or less severe PH, the time to exacerbation of dyspnea was 259.00 ± 49.87 days (mean ± SD) in 3 of 12 patients confirmed to have experienced exacerbation of dyspnea by the data obtained on the cut-off date. Proportional hazard analysis showed that the risk ratio of the drug-treated to untreated groups was 0.58, but with no significant difference noted. The time to exacerbation of dyspnea at the time of analysis was 218.17 ± 35.62 days **(**mean ± SE**)** in the untreated group and 290.71 ± 12.036 days in the drug-treated group, but with no significant difference noted. **b** Analysis of the time to an increase in the dose of O_2_ (event). Increase of the O_2_ dose: In the untreated patients with borderline or less severe PH, the time to the dose increase was 199.00 ± 132.90 days (mean ± SD) in 5 of 12 patients confirmed to have required an increase of the dose of O_2_ by the data obtained on the cutoff date. In the drug-treated patients with borderline or less severe PH, the time to the dose increase was 335.00 ± 182.43 days (mean ± SD) in 3 of 12 patients confirmed to have required an increase of the O_2_ dose based on the data obtained on the cut-off date. The risk ratio analysis showed that the hazard ratio of the drug-treated to untreated groups was 0.58. The time to O_2_ dose increase at the time of analysis was 357.71 ± 50.83 days in the untreated group and 438.20 ± 34.61 days in the drug-treated group with no significant difference between the groups, despite the results favoring the drug-treated group. In addition, only 1 drug-treated patient with borderline or less severe PH achieved a decrease of the O_2_ dose on day 243 due to an improvement of respiratory function. **c** Hospital-free survival. Of the 12 untreated patients with borderline or less severe PH, 8 were confirmed to have been hospitalized (event) by the data obtained on the cut-off date with the time to hospitalization being 241.50 ± 192.24 days (mean ± SD). Of the 12 drug-treated patients with borderline or less severe PH, 2 was confirmed to have been hospitalized by the data obtained on the cut-off date with the time to hospitalization being 239.002 ± 169.00 days. At the time of survival time analysis, hospital-free survival in the untreated group was 358.87 ± 68.65 days (mean ± SE) (median, 331 days), which was shown to be significantly different from that in the drug-treated group (603.44 ± 50.074) by proportional hazard analysis (hazard ratio [HR] of the drug-treated to untreated groups, 0.10; *P* = 0.017). **d** Overall survival. Of the 12 untreated patients with borderline or less severe PH, 7 were confirmed dead (event) by the data obtained on the cut-off date with the time to event being 309.29 ± 195.13 days (mean ± SD); of the drug-treated patients with borderline or less severe PH, 1 was confirmed dead by the data on the cut-off date with the time to event being 671 days. At the time of survival analysis, the time to event in the untreated group was 433.78 ± 66.98 days (mean ± SE), which was significantly different from that in the drug-treated group by proportional hazard analysis (HR of the drug-treated to untreated groups, 0.10; *P* = 0.0082)

#### Increase of O_2_ dose (Table 2, Figure 2b)

Of the 12 untreated patients with borderline or less severe PH, 5 were confirmed to have required an increase of the O_2_ dose based on the data obtained on the cut-off date. Of the 12 drug-treated patients with borderline or less severe PH, 3 were confirmed to have required an increase of the O_2_ dose based on the data obtained on the cutoff date. The risk ratio analysis showed that the hazard ratio of the drug-treated group to the untreated group was 0.58, with the time to O_2_ dose increase at the time of analysis being 357.71 ± 50.83 days in the untreated group versus 438.20 ± 34.61 days in the drug-treated group, which was not significantly different despite the fact that the results favored the drug-treated group. Only 1 patient with borderline or less severe PH in the drug-treated group achieved a decrease of the O_2_ dose on day 243 because of improved respiratory function.

#### Hospital-free survival (Table 2, Figure 2c)

Of the 12 untreated patients with borderline or less severe PH, 8 were confirmed to have been hospitalized based on the data obtained on the cut-off date.

In contrast, of the 12 drug-treated patients with borderline or less severe PH, 2 were confirmed to have been hospitalized based on the data obtained on the cutoff date.

At the time of survival time analysis, hospital-free survival was 358.87 ± 68.65 days (mean ± SE) (median, 331 days) in the untreated group, which was significantly different from that in the drug-treated group (603.44 ± 50.074 days) as assessed by proportional hazard analysis (hazard ratio of the drug-treated group to the untreated group, 0.19, *P* = 0.017; log-rank test, *P* = 0.019; and Wilcoxon test, *P* = 0.014).

#### Overall survival (Table 2, Figure 2d)

Of the 12 untreated patients with borderline or less severe PH, 7 were confirmed dead (event) based on the data obtained on the cut-off date. Of the 12 drug-treated patients with borderline or less severe PH, 1 was confirmed dead with the time to event being 671 days.

At the time of survival analysis, the time to event was 433.78 ± 66.98 days (mean ± SE) in the untreated group, which was shown to be significantly different from that in the drug-treated group as assessed by proportional hazard analysis (hazard ratio of the drug-treated group to the untreated group, 0.10, *P* = 0.0082; log-rank test, *P* = 0.011; and Wilcoxon test, *P* = 0.011).

#### Clinical course

Eight of the 12 untreated patients received LTOT. Of the 12 patients, 1 completed the 2-year treatment period, 2 were still on the study (with one having completed regular examinations up to month 12 and the other up to month 18), and 1 was not available for the periodic assessments from month 18 onwards due to change of the attending physician after change of address. Of the remaining 8 patients, 7 were censored from hospital-free survival analysis due to progression of respiratory failure, including 5 and 1 who were not available for the periodic assessments other than mMRC, 6MWD and TMET from months 6 and 18 onwards, respectively, and were later confirmed dead. One patient was confirmed alive at the time of analysis but was not available for the periodic assessments other than mMRC, 6MWD and TMET from month 12 onwards. The remaining 1 patient developed ileus and was confirmed to have died due to a disease other than lung disease at another hospital and was completely excluded from the periodic assessments from month 6 onwards.

As for the periodic assessments with mMRC, TMET and 6MWT, of the 11 patients assessed by mMRC at month 6, 9 each were further assessed at month 12 and 8 were further assessed at month 18, and 7 completed the assessments at month 24, including 1 patient who was assessed at months 18 and 24 by contacting the patient’s current physician after change of address. Of the 11 patients assessed by TMET and 6MWT at month 6, 8 were further assessed at month 12, and 7 completed the assessments at months 18 and 24.

Seven of the 12 drug-treated patients included in the analysis received LTOT. Four patients completed the study after finishing the assessments at month 48 and 1 of the remaining 8 patients withdrew from the study before month 6 due to hepatic dysfunction. Another patient withdrew from the study due to lung cancer detected on day 518. The last two patients were censored from hospital-free survival analysis due to exacerbation of respiratory failure on days 641 and 303, respectively, with the former confirmed dead on day 671. The remaining 4 patients were still on the study treatment (with 1 having completed regular examinations at baseline alone, 1 up to month 6, 2 up to month 12, and 1 up to month 18).

### Lung function and RHC

#### Drug-treated patients with borderline or less severe PH

Compared with baseline (Table 1b), significant changes were noted in lung function %DLCO at months 6 and 12 (month 6, +7.011, *P* = 0.010; month 12, +12.18, *P* = 0.0025) (See Additional file 9: Figure %DLCO).

Compared with baseline (Table 2b), there was a decreasing trend in mPAP at months 6 and 12 although no significant difference was noted (month 6, −2.60, *P* = 0.098, *R* = 0.84; month 12, −1.71, *P* = 0.38, *R* = 0.83). A similar trend was observed for PVR (month 6, −0.69, *P* = 0.11, *R* = 0.88; month 12, −0.41, *P* = 0.41, *R* = 0.87). Compared with baseline, there was a significant improvement in mixed venous saturation of oxygen at month 6 (+4.78, *P* = 0.037, *R* = 0.45), but no significant change was noted from baseline to month 12.

**Fig. 3:**
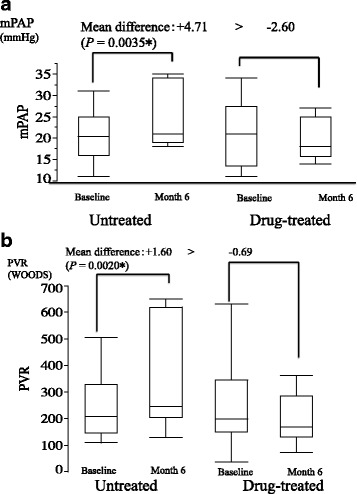
Results of RHC. **a** Comparison of changes in mPAP from baseline to month 6 between untreated and drug-treated patients with borderline or less severe PH. **b** Comparison of change in PVR from baseline to month 6 between untreated and drug-treated patients with borderline or less severe PH. Significant differences were seen between the untreated and drug-treated patients with borderline or less severe PH with regard to changes in mPAP, and PVR from baseline to month 6 (untreated vs. drug-treated: mean difference in mPAP, 4.71 vs. -2.60 mmHg, *P* = 0.0035; PVR, +1.60 vs. -0.69 woods, *P* = 0.0020)

Moreover, significant differences were observed in the drug-treated patients with borderline or less severe PH with regard to changes in mPAP, PVR and PVRI from baseline to month 6 (untreated vs. drug-treated: mPAP, +4.71 vs. -2.60 mmHg, *P* = 0.0035; PVR, +1.60 vs. -0.69 woods, *P* = 0.0020) (Fig. 3).

It will be a long time, however, before comparisons of data can be made between the groups for month 12 onwards, with many untreated patients with borderline or less severe PH having been censored from hospital-free survival analysis with an even smaller number of patients available for the periodic assessments.

Results for other assessment parameters (See Additional file 10, Additional file 11: Figure ADL, Additional file 12: Figure TTE, and Additional file 13: Figure Arterial blood analysis).

Overall, while drug-treated patients with borderline or less severe PH tended to fare better than untreated patients with borderline or less severe PH, it was difficult to draw any conclusion due to the small number of patients currently available for analysis, especially in untreated patients with borderline or less severe PH.

Thus, while the study appears to provide potentially valuable findings at this stage, their relevance and/or validity require to be closely examined when the final data of this trial become available.

## Discussion

Many patients with IPF experience a rapid elevation of PAP as well as progression of IPF [7] and elevated PAP is shown to be associated with poor prognosis [7]. Therapies currently available for slowing the progression of fibrosis, such as pirfenidone, cannot be expected to improve IPF [30]. Besides, for any honeycomb lung that has become completely organized, no realistic treatment options are available, other than symptomatic relief with LTOT, to neutralize the progression of respiratory failure.

In addition, it remains largely unclear how IPF and associated PH may interact to influence each other. Again, in agreement with the ATS/ERS/JRS/ALAT Clinical Practice Guideline 2015 that remains inconclusive with regard to the effect of drugs for PAH on IPH except in a subset of cases, recommending dual ERAs in patients with IPF recommended as “worthwhile considerations” and bosentan as “a conditional recommendation against use” [31], our study provided no definite clue as to how IPF and associated PH may interact. Furthermore, while our study did not allow PH and lung fibrosis to be examined for any relationship due to its small sample size, it did show no significant correlation between FVC and mPAP, suggesting that how PH and lung fibrosis interact may not readily lend itself to clarification.

Against this background, bosentan was shown to be efficacious in a subset of IIP patients in the BULD-1 study, and this is in contrast to the results of a number of randomized controlled trials [10, 32, 33], including the BUILD-3 and ARTEMIS trials, conducted in a wide range of biopsy-proven IPF patients (where the pathology of IPF studied, including the pathologic activity, varied widely, i.e., those with fibrotic IIP, f-NSIP, elevated PAP and decreased CI), which argued against the use of bosentan in patients with PH and f-IIP. Of note, these studies failed to evaluate bosentan in patients perfectly matched for background IPF, suggesting that early intervention with bosentan in those with mildly elevated PAP may not have been sufficiently explored, while the patient background factors may have additively or synergistically contributed to the unfavorable outcomes reported in these studies.

With these considerations in mind, we enrolled patients with completely organized honeycomb lung in this study to ensure that all study subjects were as nearly matched for IPF background as possible and that they had pathologically inactive IPF (i.e., they had no active inflammatory lesion, such as GGO) that would require no change of treatment during the study. Since the subjects of this study were required to regularly visit our hospital, patients who presented to our hospital for the first time with symptoms of progressive respiratory failure were enrolled (those without symptoms of progressive respiratory failure were not eligible for treatment at regular intervals at our hospital and were followed up at some other nearby hospital).

The present study included IPF patients with completely organized pulmonary fibrosis alone in an attempt to rule out the potential influence of IPF-associated interstitial inflammation and inter-individual differences in IPF status. As a result, the majority of patients enrolled in the study were found to have borderline to less severe PH. While it remains unclear why these patients comprised the majority, in retrospect, ambulatory patient enrollment may have led to those IPF patients who required no emergency or acute intensive care, i.e., those with only honeycomb lesions accompanied by gradually progressive dyspnea, being included in the study, who could therefore represent a selected segment of the entire IPF population. Thus, the present bosentan study was conducted in IPF patients with progressive dyspnea despite minimal IPF pulmonary lesion activity, thus ruling out the additive or synergistic influence of IPF lesions on progression of dyspnea to unequivocally demonstrate the impact of therapeutic intervention for PH in progressive dyspnea and to investigate treatment-associated pathophysiological changes in cardiac function using ECG, as well as the long-term efficacy and safety of early therapeutic intervention in PH with bosentan, with borderline PH defined as mean PAP on effort (mPAPOE) ≥ 30 mmHg (including mPAP at rest <25 mmHg).

At its early phase involving a small number of patients, this study demonstrated a greater-than-expected significant difference in prognosis between bosentan-treated and untreated patients with progressive respiratory failure who were confirmed to have completely organized honeycomb lung. Based on this finding, we performed an ad hoc interim analysis, which demonstrated that bosentan therapy led to a clearly better prognosis in patients with honeycomb lung suffering from symptoms of progressive respiratory failure than that in untreated patients, which was very poor. At the same time, study results reconfirmed that patients with progressive respiratory failure with completely organized honeycomb lung have a really poor prognosis. Although the possible imbalance in patient characteristics between the two groups may have affected the study results, this study adhered to randomization with the envelope method, thus making such possibility rather unlikely.

The main limitation of this study is the small number of patients included in the analysis, but this interim report was prepared following detection of a significant difference in prognosis in patients with borderline or less severe PH treated with bosentan when only half the number of patients targeted had completed the study. Again, with this study still at an exploratory stage, we have no sufficient information to determine the sample size, but an earlier bosentan repeated-dose study (AC-052-111 trial) of patients with PAH (WHO functional class III or above) conducted in Japan provided the rationale for the sample size required (i.e., 11 patients required to conduct a two-sided *t*-test for AUC with two-sided significance level of 5% and 90% power). Given the current state of clinical trials in this field, the sample size of this study appears to be never too small. It is highly likely that the favorable prognosis seen in the bosentan-treated patients with borderline or less severe PH might have been affected by inclusion of those with IPF experiencing a rapid elevation of PAP (not always seen among the untreated patients with borderline or less severe PH undergoing the periodic assessments). It is also possible, however, that rapid PAP elevation may have led to many patients being excluded from periodic assessments, while slowly progressive PAP elevation may have allowed patients to undergo the periodic assessments, although it is difficult to prove one way or the other.

Despite this limitation, however, our study has raised the following possibilities. First, targeting patients with completely organized honeycomb lung and symptoms of progressive respiratory failure might help select patients with rapid PAP elevation. Second, the use of bosentan may be associated with improved prognosis in selected patients similar to those included in this study. Moreover, although previous reports have failed to demonstrate a significant difference in prognosis in the entire IPF patient population with any of the drugs tested, as therapeutic options capable of suppressing the pathology of IPF become available in the future, the use of bosentan in combination with any such option might contribute further to improvements in prognosis.

In addition, while the study data remain yet to mature at present, the available data demonstrate improvements in exercise tolerance over time in bosentan-treated patients compared with untreated patients thus favoring bosentan therapy, although many untreated patients were censored from hospital-free survival analysis and excluded from the periodic assessments. If these effects on exercise tolerance can be replicated through accumulation of data from continuation of this study, bosentan may have a role to play in protecting against declines in exercise tolerance by working at various levels. Moreover, changes in respiratory conditions among the drug-treated patients suggest that bosentan therapy may have corrected abnormal breath patterns that tended toward hyperventilation. These results suggest a potential role for bosentan in delaying the progression of respiratory failure and that a decrease in respiratory rate from relief of dyspnea may be linked to a trend toward increased PO_2_, decreased PCO_2_ and increased PH after exercise tolerance and stress testing, which corresponded to improvements seen in breathing efficiency after stress testing, despite no significant change in 6MWD in which the patients were assessed at the pace each patient felt comfortable. Otherwise, while the study data also suggest improvements in RV function on TTE in patients with borderline or less severe PH treated with bosentan, this finding requires to be examined at the completion of the study (see Additional file 10).

Again, while the drug-treated group did not require LTOT or increasing the O_2_ inhalation dose, the untreated group reached the endpoint of hospital survival in a very short time with progressive dyspnea.

Another limitation of the study is that, among the exercises leading to increased PAP during RHC, it included clasping and opening both hands repeatedly with a full strain exerted on the body, which allowed PAP to be monitored but made it rather difficult to continue the exercise during CO measurement thus making PVR, the most critical of all parameters, less amenable to measurement. While the reasons for the observed increases in PAP during exercise in the study can only be surmised, they may include changes in PAP or increases in pulmonary blood flow associated with PVR during exercise [34–36] and intrathoracic pressure associated with straining on the part of the patients being evaluated.

A still further limitation of the study is that all patients with lower values of PAP (mPAP <25 or mPAPOE <30) were included in the untreated group, while all patients should have been randomized. Health insurance in Japan made it hardly feasible, however, to design this study as a randomized trial in which patients without PH would also be randomized to bosentan or any other drug specific for PAH. Again, given that patients with IPF might show variable outcomes, e.g., rapid declines, frequent exacerbations, or slow declines, thus representing a heterogeneous population, patients may have had to be enrolled only after their lung function has been shown to be stable for many years. However, it was simply unfeasible to enroll patients only after their lung function had been shown to be stable for some time. Instead, we have further refined the definition of eligible patients as those confirmed to have completely organized honeycomb lung and to have no active inflammatory lesion, such as GGO.

In addition, the inclusion criteria initially defined the patient age as ranging between 20 and 40 years old to exclude familial pulmonary fibrosis. Given that this could also exclude IPF, however, the patient age may have been better defined as 40 years old or older and may have led to different outcomes. Indeed, a retrospective analysis of all patients enrolled confirmed that they ranged in age between 51 and 80 years old, which has led us to redefine the patient age as 40 years old or older.

While all patients confirmed to have no progressive pulmonary fibrosis on CT were given detailed explanations as to the potential adverse effects associated with the use of antifibrotics, such as pirfenidone or nitentanib, which has only recently been launched in Japan and indicated for very few patients, as well as the costs due under current health insurance, none of these patients had previously received any medical treatment for IPF within 3 months prior to their visit and none wished to receive any antifibrotic after the first 3 months or later. Thus, further study is warranted to investigate whether various treatment options, including combination therapy with bosentan and an antifibrotic, may lead to further improvements in prognosis in these patients.

## Conclusions

This was an interim report of our ongoing long-term study conducted to evaluate the effects of bosentan, as a PAH-specific drug, on IPF-associated PH based on detailed data analysis. Despite its limitations, the study appears to suggest that the bosentan-treated group fared remarkably better than the untreated group, while it was thought likely that those without borderline PH or PH receiving no treatment were associated with poor prognosis, and those with borderline PH or PH receiving bosentan therapy were associated with better prognosis. Again, study findings suggest that there exists a subset of IPF patients who might benefit from bosentan therapy with regard to improvements in IPF and prognosis. The authors plan to prepare a final report after accrual of further patients required to complete the study.

## Additional files

Additional file 1: Supplementary document on subgroup analysis. Supplementary document on patient grouping. (DOCX 12 kb)

Additional file 2: Supplementary data on determination of sample size. Data included as a basis for sample size determination in this study. (DOCX 17 kb)

Additional file 3: Supplementary document on parameters. Parameters included for evaluation in this study. (DOCX 19 kb)

Additional file 4: Figure S1 Methods for and results of Doppler measurements performed in this study. (PPT 154 kb)

Additional file 5: Figure S2 TMET (Treadmill exercise test) protocol as part of the supplementary document on parameters. (PPTX 54 kb)

Additional file 6: Figure S3 Schedule for evaluation of parameters in this study. (PDF 1240 kb)

Additional file 7: Supplementary information on Guidance for Tracleer Tablets® dosage modification. Guidelines for bosentan dose modification as applied in Japan and used in this study. (DOCX 14 kb)

Additional file 8: Supplementary data on the criteria for discontinuation of the study in individual patients. Criteria for study discontinuation in individual patients used in this study. (DOCX 16 kb)

Additional file 9: Assessment of time-course changes in %DLCO in Drug-treated patients with borderline or less severe PH. A summary of results for %DLCO. (PPTX 117 kb)

Additional file 10: Supplementary results for other parameters. A summary of results for other parameters in this study. (DOCX 18 kb)

Additional file 11: Figure ADL. Comparison of changes in mMRC between drug-treated and untreated patients with borderline or less severe PH; Comparison of changes in TMET between drug-treated and untreated patients with borderline PH or less severe PH. (PPTX 190 kb)

Additional file 12: Figure TTE. Change in PA AcT from baseline to month 12 in drug-treated patients with borderline or less severe PH. (PPTX 61 kb)

Additional file 13: Figure Arterial blood analysis. a. Time-course change in PaO_2_ at rest in drug-treated patients with borderline or less severe PH; b. Change in post 6MWT aortic pH in drug-treated patients with borderline PH or less severe PH. (PPTX 225 kb)

Additional file 14: Supplementary data on procedures regarding informed consent. Procedures for informed consent. (DOCX 14 kb)

Additional file 15: Supplementary information regarding compensation in case of trial-related injury or death. Compensation or indemnity for injury or death. Scheme for compensation or indemnity for injury or death in this study. (DOCX 14 kb)

Additional file 16: Supplementary information regarding medical expenses. Medical expenses anticipated in this study including those to be borne by subjects. (DOCX 14 kb)

## Abbreviations

6MWT: 6-min walk test
ABG: Arterial blood gas
ADL: Activities of daily living
BNP: Brain natriuretic peptide
CCO: Continuous cardiac output
CI: Cardiac index
CO: Cardiac output
CT: Computed tomography
DPAOE: Diastolic pulmonary arterial pressure on effort
ECG: Echocardiography
f-IIP: Fibrotic idiopathic interstitial pneumonia
f-NSIP: Fibrotic nonspecific interstitial pneumonia
HPV: Hypoxic pulmonary vasoconstriction
IPF: Idiopathic pulmonary fibrosis
LTOT: Long-term oxygen therapy
mPAP: Mean pulmonary arterial pressure
mPAPOE: Mean pulmonary arterial pressure on effort
PAH: Pulmonary arterial hypertension
PaO_2_: Partial pressure of oxygen in arterial blood
PAP: Pulmonary arterial pressure
PAWP: Pulmonary capillary wedge pressure
PDE-5: Phosphodiesterase type 5
PH: Pulmonary hypertension
PVR: Pulmonary vascular resistance
RAP: Right arterial pressure
RVP: Right ventricular pressure
SPAPOE: Systolic pulmonary arterial pressure on effort
