# Supplementary material for: Potential benefit of bosentan therapy in borderline or less severe pulmonary hypertension secondary to idiopathic pulmonary fibrosis—an interim analysis of results from a prospective, single-center, randomized, parallel-group study
Source: BMC Pulm Med. 2017 Dec 13;17:200. doi: 10.1186/s12890-017-0523-2 (PMC5729252; doi:10.1186/s12890-017-0523-2)
Supplement: Supplementary file 1 — Supplementary document on subgroup analysis. Supplementary document on patient grouping. (DOCX 12 kb) [file 12890_2017_523_MOESM1_ESM.docx]

***Drug-treated group***

(1) Patients with pure organized honeycomb lung and severe PH (mPAP at rest ≥ 35 mmHg) due to concurrent IPF: 20 patients

(2) Patients with pure organized honeycomb lung and borderline or less severe PH (mPAP at rest < 35 mmHg and, 25 mmHg ≤ mPAP at rest and/or mPAPOE ≥ 30 mmHg): 20 patients

***Untreated group***

(1) Patients with pure organized honeycomb lung and severe PH (mPAP at rest ≥ 35 mmHg): 20 patients

(2) Patients with pure organized honeycomb lung and borderline or less severe PH (mPAP at rest < 35 mmHg with 25 mmHg ≤ mPAP at rest and/or mPAPOE ≥ 30 mmHg): 20 patients

(3) Patients with pure organized honeycomb lung but without borderline PH or PH (non-PH: mPAP at rest < 25 mmHg and mPAPOE < 30 mmHg) and impaired right heart function: 20 patients

(4) Patients with pure organized honeycomb lung but without borderline PH or PH (non-PH: mPAP at rest < 25 mmHg and mPAPOE < 30 mmHg) or impaired right heart function: 20 patients

The study required that IPF patients be randomized to drug-treated and untreated groups to investigate their clinical course in real-world settings, with no change of treatment allowed including bosentan for 2 years or until their death as a rule, except for minimal symptomatic therapy including oxygen volume adjustments that met none of the exclusion criteria.
