# Supplementary material for: Potential benefit of bosentan therapy in borderline or less severe pulmonary hypertension secondary to idiopathic pulmonary fibrosis—an interim analysis of results from a prospective, single-center, randomized, parallel-group study
Source: BMC Pulm Med. 2017 Dec 13;17:200. doi: 10.1186/s12890-017-0523-2 (PMC5729252; doi:10.1186/s12890-017-0523-2)
Supplement: Supplementary file 2 — Supplementary data on determination of sample size. Data included as a basis for sample size determination in this study. (DOCX 17 kb) [file 12890_2017_523_MOESM2_ESM.docx]

**Supplementary data on determination of sample size**

With this study still at an exploratory stage, no sufficient information is available to determine the sample size. However, in a previous clinical study conducted in Japan (“Effects of the endothelin receptor antagonist bosentan on hemodynamics, symptoms and functional capacity in Japanese patients with severe pulmonary hypertension” by Sasayama S, et al) (Circ J. 2005;69:131-137), the change in pulmonary vascular resistance from baseline was 382 dyn.s/cm5 in the Tracleer group, with a variance of 717 and 520 dyn.s/cm5 before and after the study treatment, respectively. Assuming a correlation of 0.5 before and after the treatment, the variance estimate of change was 443 dyn.s/cm5. Assuming the change of 0 from baseline for the untreated group, at least17 patients were required in each group to compare this change between groups with 80% power using a t-test. Assuming data dropouts, 20 patients were selected for each group. We determined the sample size of this study using this information.

Further, in a repeated-dose study of bosentan (AC-052-111 trial) in patients with PAH (WHO functional class III or above) conducted in Japan, 11 patients were required to perform a two-sided t-test (two-sided significance level of 5%, 90% power) for AUC. In light of this information, we prepared this interim report based on the number of patients currently available for analysis because an obvious significant difference was already observed in prognosis between the groups compared in this study.
