# Supplementary figures and images for: Potential benefit of bosentan therapy in borderline or less severe pulmonary hypertension secondary to idiopathic pulmonary fibrosis—an interim analysis of results from a prospective, single-center, randomized, parallel-group study
Source: BMC Pulm Med. 2017 Dec 13;17:200. doi: 10.1186/s12890-017-0523-2 (PMC5729252; doi:10.1186/s12890-017-0523-2)

## Slide 1
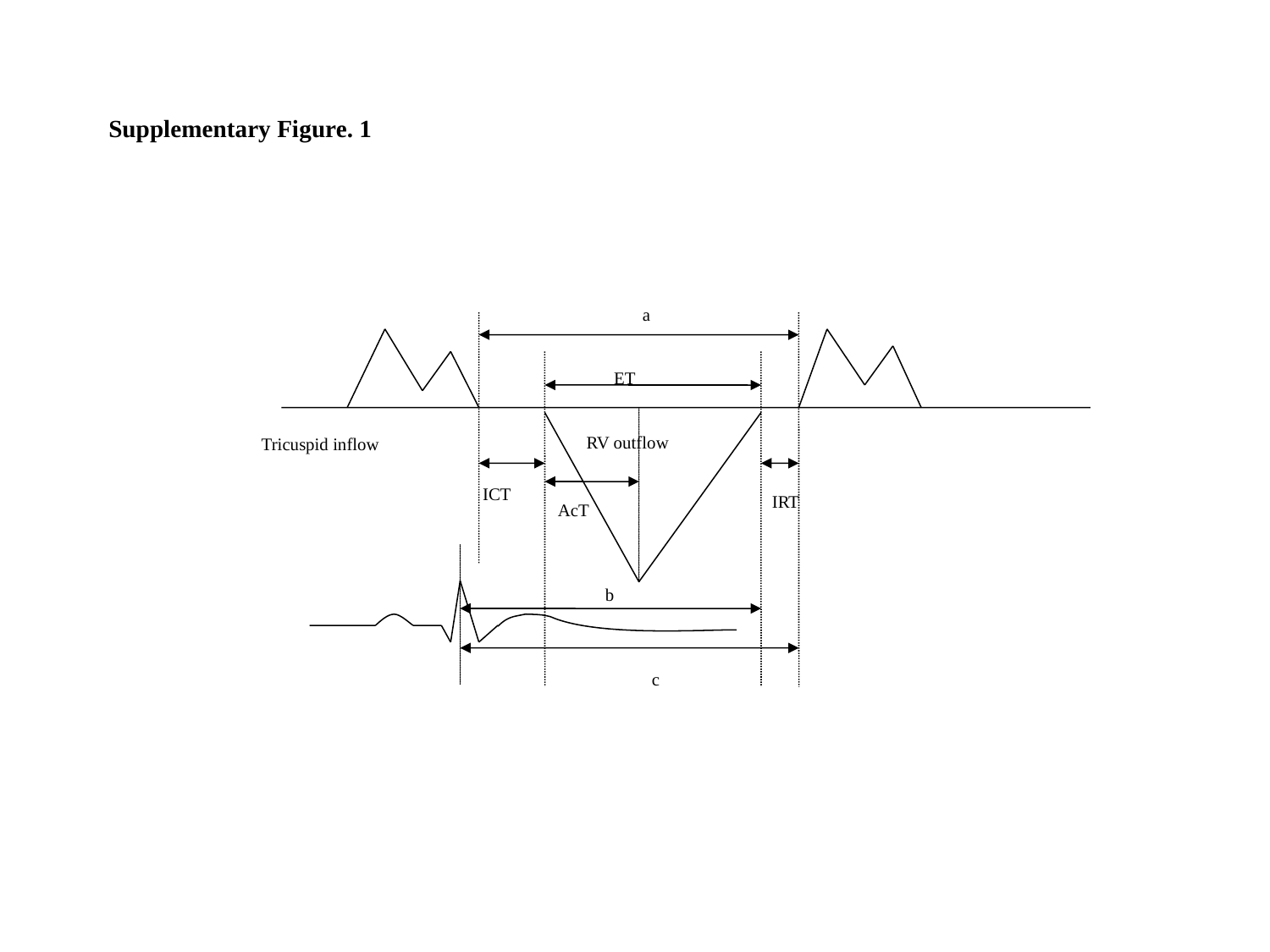

Supplementary Figure. 1
a
ET
RV outflow
Tricuspid inflow
ICT
IRT
AcT
b
c

Supplement: Supplementary file 4 — Figure S1 Methods for and results of Doppler measurements performed in this study. (PPT 154 kb) [file 12890_2017_523_MOESM4_ESM.ppt]

## Slide 1
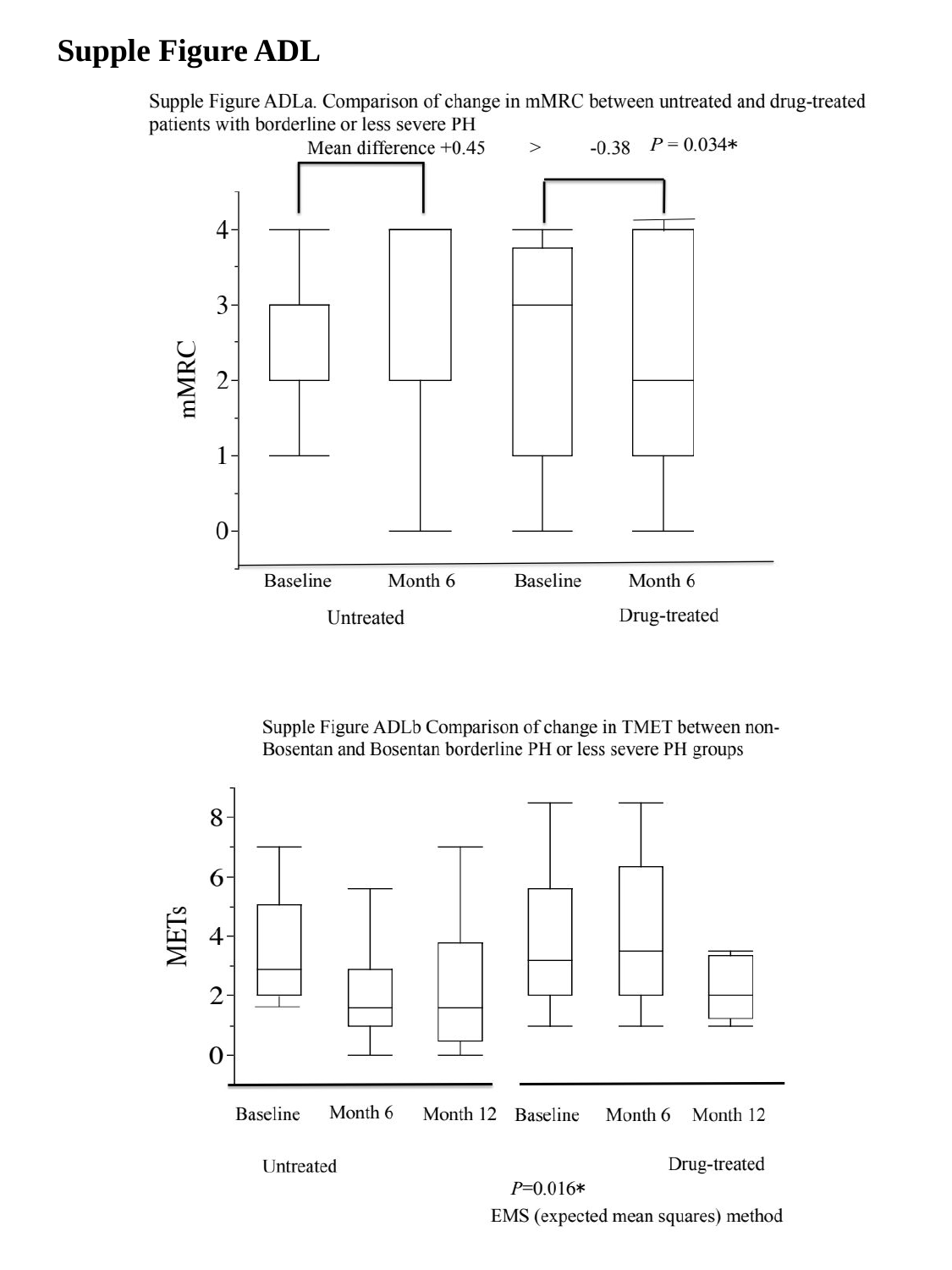

Supple Figure ADL

Supplement: Supplementary file 11 — Figure ADL. Comparison of changes in mMRC between drug-treated and untreated patients with borderline or less severe PH; Comparison of changes in TMET between drug-treated and untreated patients with borderline PH or less severe PH. (PPTX 190 kb) [file 12890_2017_523_MOESM11_ESM.pptx]

## Slide 1
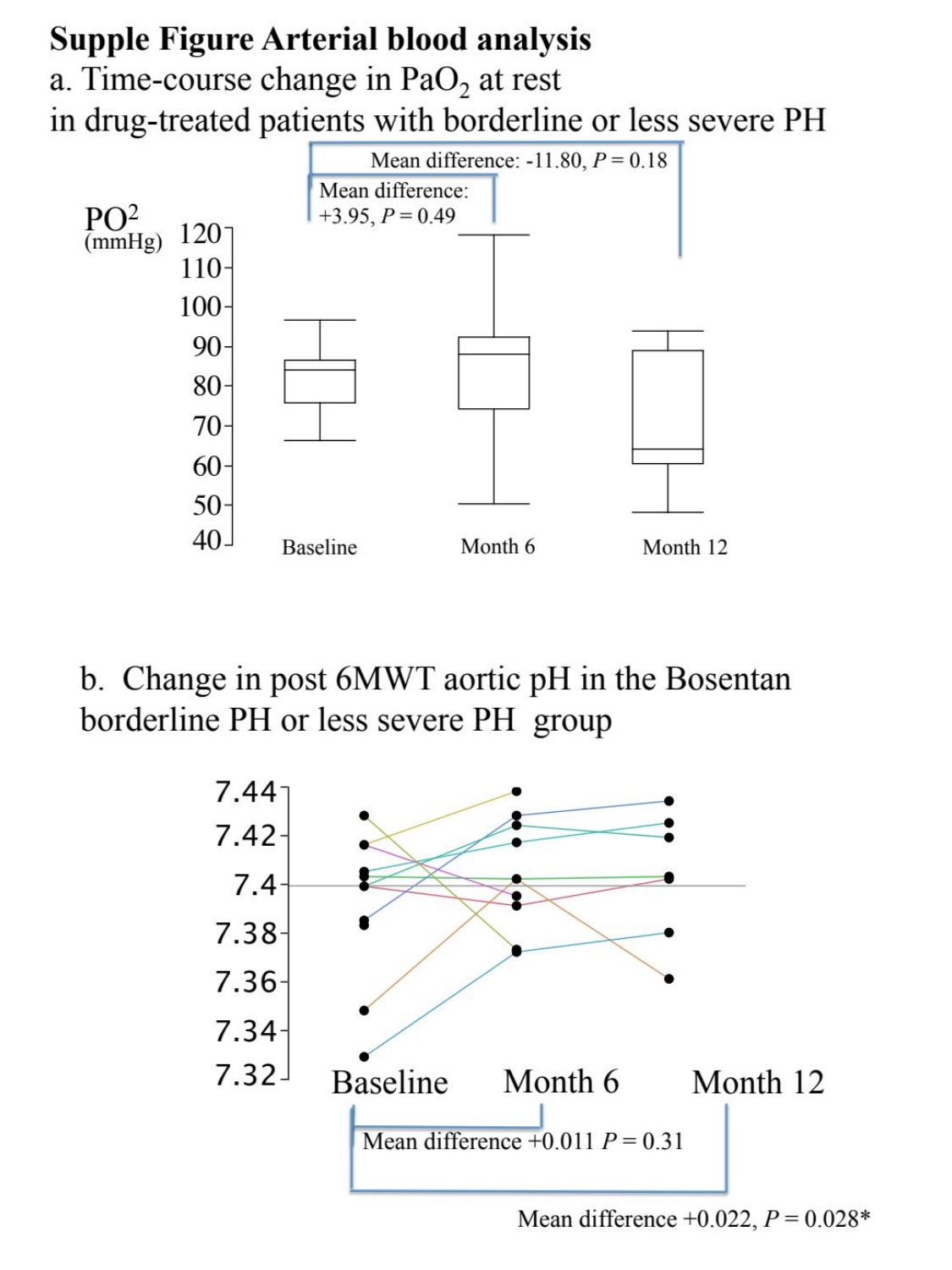

Supplement: Supplementary file 13 — Figure Arterial blood analysis. a. Time-course change in PaO2 at rest in drug-treated patients with borderline or less severe PH; b. Change in post 6MWT aortic pH in drug-treated patients with borderline PH or less severe PH. (PPTX 225 kb) [file 12890_2017_523_MOESM13_ESM.pptx]
