# Supplementary material for: Potential benefit of bosentan therapy in borderline or less severe pulmonary hypertension secondary to idiopathic pulmonary fibrosis—an interim analysis of results from a prospective, single-center, randomized, parallel-group study
Source: BMC Pulm Med. 2017 Dec 13;17:200. doi: 10.1186/s12890-017-0523-2 (PMC5729252; doi:10.1186/s12890-017-0523-2)
Supplement: Supplementary file 5 — Figure S2 TMET (Treadmill exercise test) protocol as part of the supplementary document on parameters. (PPTX 54 kb) [file 12890_2017_523_MOESM5_ESM.pptx]

## Slide 1
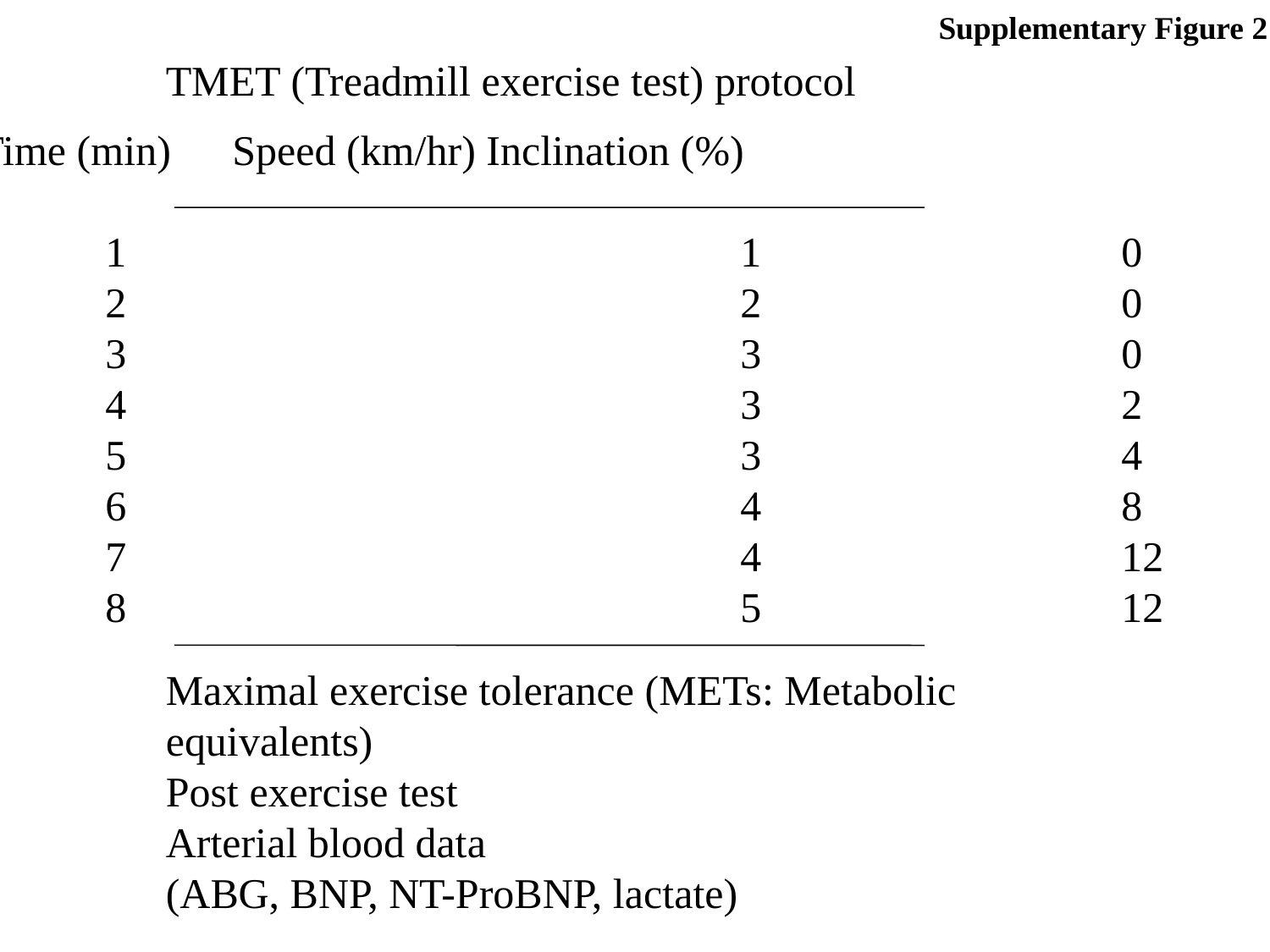

Supplementary Figure 2
TMET (Treadmill exercise test) protocol
Maximal exercise tolerance (METs: Metabolic equivalents)
Post exercise test
Arterial blood data
(ABG, BNP, NT-ProBNP, lactate)
Time (min) 	Speed (km/hr)	Inclination (%)
	1					1		 	0
	2					2		 	0
	3					3		 	0
	4					3		 	2
	5					3			4
	6					4		 	8
	7					4			12
	8					5			12
