# Supplementary material for: Potential benefit of bosentan therapy in borderline or less severe pulmonary hypertension secondary to idiopathic pulmonary fibrosis—an interim analysis of results from a prospective, single-center, randomized, parallel-group study
Source: BMC Pulm Med. 2017 Dec 13;17:200. doi: 10.1186/s12890-017-0523-2 (PMC5729252; doi:10.1186/s12890-017-0523-2)
Supplement: Supplementary file 6 — Figure S3 Schedule for evaluation of parameters in this study. (PDF 1240 kb) [file 12890_2017_523_MOESM6_ESM.pdf]

Supplementary Figure 3

Item		Run-in period	Start of treatment	Treatment period (24 months)					
Time							Week 8 onwards		
		Weeks -0 to -2	Week 0 (For untreated group, 2 weeks after RHC)	(Week 1)	Week 2	Week 4	Every 4 weeks	Every 6 months	At completion (discontinuation)
Informed consent		○							
Patient background		○							
Study drug administration (Drug to treat PAH)*			←						→
Subjective and objective symptoms		○	○	○	○	○	○	○	○
Monitoring for adverse events			←						→
Blood pressure		○			○*	○	○	○	○
Pulse rate		○			○*	○	○	○	○
Body weight		○						○	○
Laboratory tests	Hematology	○			○*	○*	○	○	○
	Biochemistry (NT-proBNP, etc.)	○			○*	○*	○	○	○
	Urinalysis	○				○*	○	○	○
Chest X-ray		○				○	○	○	○
ECG		○						○	
Echocardiography		○						○	○
RHC		○						○	
ADL		○	○					○	○
Exercise stress testing		○						○	○
PFT		○						○	○
Arterial blood analysis		○						○	○

*To be conducted only in patients given the study drug
