# Supplementary material for: Potential benefit of bosentan therapy in borderline or less severe pulmonary hypertension secondary to idiopathic pulmonary fibrosis—an interim analysis of results from a prospective, single-center, randomized, parallel-group study
Source: BMC Pulm Med. 2017 Dec 13;17:200. doi: 10.1186/s12890-017-0523-2 (PMC5729252; doi:10.1186/s12890-017-0523-2)
Supplement: Supplementary file 7 — Supplementary information on Guidance for Tracleer Tablets® dosage modification. Guidelines for bosentan dose modification as applied in Japan and used in this study. (DOCX 14 kb) [file 12890_2017_523_MOESM7_ESM.docx]

**Supplementary information on Guidance for Tracleer Tablets® dosage modification**

If a patient receiving Tracleer experiences an AST (GOT) or ALT (GPT) elevation greater than 3-fold the upper limit of the normal reference range, dosage modification and liver function tests will be conducted with reference to the guidance provided below:

AST (GOT)/ ALT (GPT): Action taken with Tracleer therapy and frequency of liver function tests

>3-fold and ≤ 5-fold ULN The dosage of Tracleer should be reduced or the therapy with the drug discontinued. Thereafter, AST and ALT should be measured at least every 2 weeks. If the values return to baseline, Tracleer therapy may be continued or resumed as appropriate.*

>5-fold and ≤8-fold ULN Tracleer therapy should be discontinued. Thereafter, AST and ALT should be measured at least every 2 weeks. If the values return to baseline, resumption of Tracleer therapy may be considered.*

>8-fold ULN Tracleer therapy should be discontinued and should not be resumed.

ULN: Upper limit of normal

* To resume Tracleer therapy, the starting dose should be used. After the therapy is resumed, AST and ALT values should be measured again within 3 days, and then at 2 weeks from the date the therapy is resumed. Subsequently, the therapy is given again in accordance with the above guidance for dosage adjustment and liver function tests.
