# Supplementary material for: Potential benefit of bosentan therapy in borderline or less severe pulmonary hypertension secondary to idiopathic pulmonary fibrosis—an interim analysis of results from a prospective, single-center, randomized, parallel-group study
Source: BMC Pulm Med. 2017 Dec 13;17:200. doi: 10.1186/s12890-017-0523-2 (PMC5729252; doi:10.1186/s12890-017-0523-2)
Supplement: Supplementary file 8 — Supplementary data on the criteria for discontinuation of the study in individual patients. Criteria for study discontinuation in individual patients used in this study. (DOCX 16 kb) [file 12890_2017_523_MOESM8_ESM.docx]

**Supplementary data on the criteria for discontinuation of the study in individual patients**

In any of the following instances, the study will be discontinued in individual patients:

1) If the patient withdraws consent;

2) If the patient requires discontinuation of treatment due to aggravation of the primary disease. (as a rule, appropriate therapy for the primary lung disease, including a change in the dose of O_2_, should be allowed according to treatment guidelines);

3) If the patient requests change or discontinuation of treatment;

4) If the patient becomes pregnant; or

5) If the attending physician judges that the patient has difficulty continuing the study;

In instances “1)” and “3),” the data obtained up to the date of discontinuation will not be used for the purpose of the study.

In other instances, the data obtained up to the date of discontinuation will be used for the purpose of the study.

In either of the following instances, the study may be discontinued in individual patients permanently or temporarily:

1) If the patient experiences an AST (GOT) or ALT (GPT) elevation greater than 3-fold the upper limit of the normal reference range; or

2) If the patient experiences a serious adverse event.
