# Supplementary material for: Potential benefit of bosentan therapy in borderline or less severe pulmonary hypertension secondary to idiopathic pulmonary fibrosis—an interim analysis of results from a prospective, single-center, randomized, parallel-group study
Source: BMC Pulm Med. 2017 Dec 13;17:200. doi: 10.1186/s12890-017-0523-2 (PMC5729252; doi:10.1186/s12890-017-0523-2)
Supplement: Supplementary file 9 — Assessment of time-course changes in %DLCO in Drug-treated patients with borderline or less severe PH. A summary of results for %DLCO. (PPTX 117 kb) [file 12890_2017_523_MOESM9_ESM.pptx]

## Slide 1
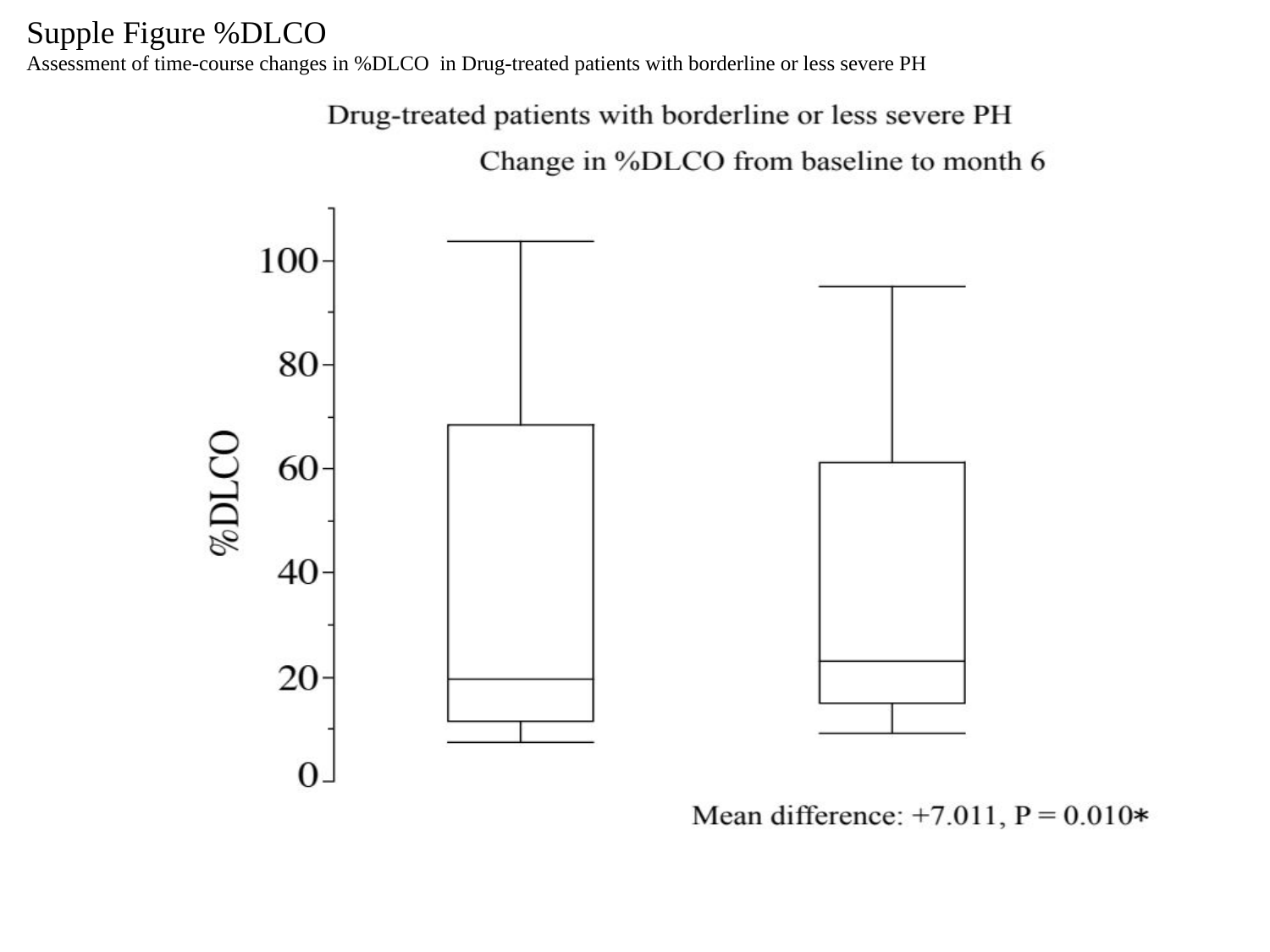

Supple Figure %DLCO
Assessment of time-course changes in %DLCO in Drug-treated patients with borderline or less severe PH
