# Supplementary material for: Potential benefit of bosentan therapy in borderline or less severe pulmonary hypertension secondary to idiopathic pulmonary fibrosis—an interim analysis of results from a prospective, single-center, randomized, parallel-group study
Source: BMC Pulm Med. 2017 Dec 13;17:200. doi: 10.1186/s12890-017-0523-2 (PMC5729252; doi:10.1186/s12890-017-0523-2)
Supplement: Supplementary file 10 — Supplementary results for other parameters. A summary of results for other parameters in this study. (DOCX 18 kb) [file 12890_2017_523_MOESM10_ESM.docx]

**Supplementary results for other parameters**

**Activities of daily living (ADL)**

***Untreated patients with borderline or less severe PH***

Compared with baseline (Table 1), no significant changes were observed in any ADL parameters, excluding mMRC, TMET and 6MWD in untreated patients; given that many of these of patients had reached the endpoint of hospital-free survival and thus became unavailable for mMRC, TMET and 6MWD, it was difficult to draw any conclusion.

Compared with baseline, there was a trend toward exacerbation of mMRC dyspnea at month 6 (mean difference from baseline, +0.45, *P* = 0.096, R = 0.88) and month 12 (+0.50, *P* = 0.24, R = 0.48), but with no significant difference, while a significant exacerbation of dyspnea was observed from month 18 onwards (month 18, +1.00, *P* = 0.0072, R = 0.80; month 24, +1.00, *P* = 0.0072, R = 0.80).

TMET showed a trend toward exacerbation of ADLs at all time points from month 6 onwards, with a significant exacerbation noted at month 6 and from month 18 onwards (month 6, -1.36, *P* = 0.038; month 12, -1.72, *P* = 0.027; month 18, -3.23, *P* = 0.010; and month 24, -3.47, *P* = 0.011).

6MWD showed a trend toward exacerbation of ADL at all time points from month 6 onwards, with a significant exacerbation observed from month 12 onwards (month 6, -104.55 m, *P* = 0.34; month 12, -126.00 m, *P* = 0.028; month 18, -230.00 m, *P* = 0.0022; and month 24, -230.00 m, *P* = 0.0022).

***Drug-treated patients with borderline or less severe PH***

Compared with baseline (Table 1), there was a decreasing trend in mMRC at months 6 and 12, which was not significantly different (month 6, -0.30, *P* = 0.19, R = 0.89 month 12, -0.63, *P* = 0.18, R = 0.42). Since many patients had yet to become available for assessment at month 18 or later, it was too early to perform analysis on the following parameters from month 18 onwards (month 18, -0.43, *P* = 0.20, R = 0.38, month24, -0.20, *P* = 0.62, R = 0.56): SGRQ symptom (month 6, -14.63, *P* = 0.083; month 12, -11.74, *P* = 0.27), SGRQ activity (month 6, -4.17, *P* = 0.47; and month 12, -6.94, *P* = 0.36), SGRQ Impact (month 6, -1.20, *P* = 0.84; month 12, -0.50, *P* = 0.94), T-SGRQ (month 6, -4.37, *P* = 0.42; month 12, -4.39, *P* = 0.42), SF36PF (month 6, -0.77, *P* = 0.92; month 12, +2.32, *P* = 0.81), SF36RP (month 6, +3.75, *P* = 0.68; month 12, -0.90, *P* = 0.94), SF36BP (month 6, -3.90, *P* = 0.52; month 12, +1.14, *P* = 0.85), SF36GH (month 6, +0.70, *P* = 0.92; month 12, +10.36, *P* = 0.21), SF36VT (month 6, -1.26, *P* = 0.88; month 12, +4.46, P = 0.71); SF36SF (month 6, -2.50, *P* = 0.83; month 12, +8.93, *P* = 0.39), SF36RE (month 6, -13.33, *P* = 0.39; month 12, -23.80, *P* = 0.18), SF36MH (month 6, +31.62, *P* < 0.0001; month 12, -13.57, *P* = 0.29), 6MWD (month 6, -14.90 m, *P* = 0.45; month 12, -82.44 m, *P* = 0.080), and TMET (month 6, +0.42 METs, *P* = 0.19; month 12, -0.44 METs, *P* = 0.27).

Moreover, the changes in SGRQ and SF36 scores from baseline to month 6 generally showed a trend for improvement in ADL in the drug-treated group compared with the untreated group, but with no significant difference noted. There was a significant difference between the drug-treated and untreated groups in the change in mMRC from baseline to month 6 (*P* = 0.034) (Supple Figure ADLa).

Of note, repeated measures analysis of TMET data based on the EMS method using the standard least squares test showed a significant difference favoring the drug-treated group in the change in maximal exercise tolerance from baseline to month 12 (*P* = 0.016) (Supple Figure ADLb).

Thus, taken together, study findings suggest that declines in maximal exercise tolerance may have been delayed in the drug-treated group compared to the untreated group.

**その他のparameters**

***Untreated patients with borderline or less severe PH***

Although it was difficult to draw any conclusion from the small number of patients currently available for analysis, no significant change was observed in any of the parameters assessed.

***Drug-treated patients with borderline or less severe PH***

**TTE**

Compared with baseline (Table 2), there was a significant change in PA AcT at month 12 (-10.71 msec, *P* = 0.013) (supple Fig. TTE). In addition, while other parameters showed no significant change from baseline, some parameters, such as RV TEI index, tended to improve.

**Laboratory data (blood sampling at rest)**

Compared with baseline (Table 1b), there were no significant changes in AGB parameters. Compared with baseline, PO_2_ was rather increased at month 6 with a mean difference of +3.95 (*P* = 0.49) although no significant difference was noted. And compared with baseline, PO_2_ at month 12 showed a decreasing trend (-11.80, *P* = 0.18) although no significant difference was noted. (Supple Figure Arterial blood analysis**.** a.). On the other hand, compared with baseline, there was a decreasing trend in pH at months 6 and 12, but with no significant difference noted (month 6, -0.0073, *P* = 0.40; month 12, -0.0081, *P* = 0.55). Again, there was a trend toward increased PCO2, likely reflecting the trends over time toward improvements in mMRC, SF36 and SGRQ as well as the resolution of hyperventilation associated with alleviation of dyspnea in PT. Similarly, compared with baseline, there were decreases in BNP and NT-ProBNP at month 6, but with no significant difference noted.

**Post-6MWT**

Compared with baseline, there was an increase in pH at month 6 and a significant increase at month 12 (month 6, +0.011, *P* = 0.31; month 12, +0.022, *P* = 0.028). Compared with baseline, there was a decreasing trend in PCO_2_ at month 12, but with no significant difference noted (month 6, -0.97, *P* = 0.42; month 12, -1.76, *P* = 0.39). There was an increasing trend in PO_2_ at months 6 and 12, but with no significant difference noted (month 6, +5.70, P = 0.44; month12, +3.27, *P* = 0.76) (Supple Figure Arterial blood analysis**.** b).

**Post-TMET**

Compared with baseline, no significant change in TMET data was noted up to month 12. The patients’ response to exercise stress varied from a more than 0.5 mM/dL increase in arterial plasma lactate after exercise to no change. Serial or time-course changes in arterial plasma lactate revealed no effect of bosentan therapy (31).
