# Supplementary material for: Potential benefit of bosentan therapy in borderline or less severe pulmonary hypertension secondary to idiopathic pulmonary fibrosis—an interim analysis of results from a prospective, single-center, randomized, parallel-group study
Source: BMC Pulm Med. 2017 Dec 13;17:200. doi: 10.1186/s12890-017-0523-2 (PMC5729252; doi:10.1186/s12890-017-0523-2)
Supplement: Supplementary file 12 — Figure TTE. Change in PA AcT from baseline to month 12 in drug-treated patients with borderline or less severe PH. (PPTX 61 kb) [file 12890_2017_523_MOESM12_ESM.pptx]

## Slide 1
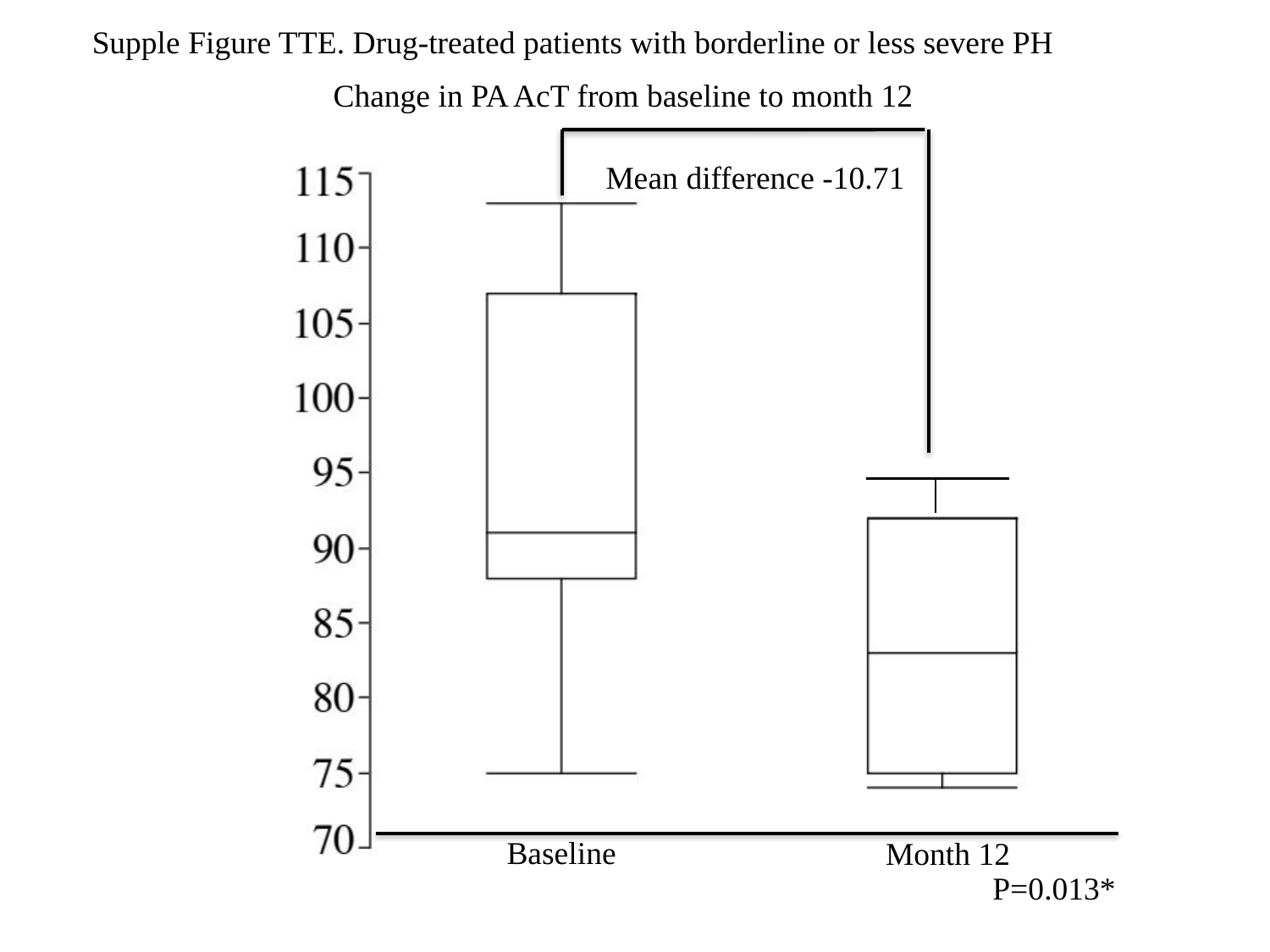

Supple Figure TTE. Drug-treated patients with borderline or less severe PH
Change in PA AcT from baseline to month 12
Mean difference -10.71
Baseline
Month 12
P=0.013*
