# Supplementary material for: Potential benefit of bosentan therapy in borderline or less severe pulmonary hypertension secondary to idiopathic pulmonary fibrosis—an interim analysis of results from a prospective, single-center, randomized, parallel-group study
Source: BMC Pulm Med. 2017 Dec 13;17:200. doi: 10.1186/s12890-017-0523-2 (PMC5729252; doi:10.1186/s12890-017-0523-2)
Supplement: Supplementary file 14 — Supplementary data on procedures regarding informed consent. Procedures for informed consent. (DOCX 14 kb) [file 12890_2017_523_MOESM14_ESM.docx]

**Supplementary data on procedures regarding informed consent**

An informed consent form describing the following items was prepared. Consent had to be obtained in writing.

1. Introduction: About target disease and Tracleer Tablets®

2. Purpose of the study

3. Study methods

4. Planned duration of your participation in this study

5. Planned number of patients participating in this study

6. Anticipated benefits and possible side effects of the study drug

7. Alternative treatment methods

8. Health injury during this study

9. Voluntary participation in this study

10. Prompt notification of information related to the study drug

11. Possible discontinuation of treatment with the study drug

12. Review of medical records during or after the study

13. Publication and protection of personal data

14. Responsibilities associated with consent to participate in this study

15. Anticipated expenses to be borne by patients

16. Study center

17. Attending physician and medical consultation
