# Supplementary material for: Potential benefit of bosentan therapy in borderline or less severe pulmonary hypertension secondary to idiopathic pulmonary fibrosis—an interim analysis of results from a prospective, single-center, randomized, parallel-group study
Source: BMC Pulm Med. 2017 Dec 13;17:200. doi: 10.1186/s12890-017-0523-2 (PMC5729252; doi:10.1186/s12890-017-0523-2)
Supplement: Supplementary file 15 — Supplementary information regarding compensation in case of trial-related injury or death. Compensation or indemnity for injury or death. Scheme for compensation or indemnity for injury or death in this study. (DOCX 14 kb) [file 12890_2017_523_MOESM15_ESM.docx]

**Supplementary information regarding compensation in case of trial-related injury or death**

**Compensation or indemnity for injury or death**

There are no special compensation arrangements. Any health injury in this study will be handled in the same manner as health injuries or medical accidents occurring in usual medical practice. If indemnity is claimed for any health injury, the indemnity will be paid within the range covered by the medical liability insurance policy bought by the hospital or privately by the physician.
