# Supplementary material for: Potential benefit of bosentan therapy in borderline or less severe pulmonary hypertension secondary to idiopathic pulmonary fibrosis—an interim analysis of results from a prospective, single-center, randomized, parallel-group study
Source: BMC Pulm Med. 2017 Dec 13;17:200. doi: 10.1186/s12890-017-0523-2 (PMC5729252; doi:10.1186/s12890-017-0523-2)
Supplement: Supplementary file 16 — Supplementary information regarding medical expenses. Medical expenses anticipated in this study including those to be borne by subjects. (DOCX 14 kb) [file 12890_2017_523_MOESM16_ESM.docx]

**Supplementary information regarding medical expenses**

**Anticipated medical expenses (borne by subjects)**

In this study, such treatment will be given to patients as is covered by usual health insurance, and patients are expected to cover the amount due to them for medical expenses as required by their health insurance. There will be no extra medical expenses for this study. In most cases, the amount due to the patients will be reimbursed by a public medical expense subsidy program (for designated diseases). There will be no payment for transportation expenses or honorarium associated with this study.
